# Hydroxychloroquine/Chloroquine as Therapeutics for COVID-19: Truth under the Mystery

**DOI:** 10.7150/ijbs.59547

**Published:** 2021-04-10

**Authors:** Yao Chen, Mei-Xiu Li, Guo-Dong Lu, Han-Ming Shen, Jing Zhou

**Affiliations:** 1 Department of Physiology, School of Preclinical Medicine, Guangxi Medical University, Nanning, Guangxi Province, China, 530021.; 2 Department of Toxicology, School of Public Health, Guangxi Medical University, Nanning, Guangxi Province, China, 530021.; 3 Faculty of Health Sciences, University of Macau, Taipa, Macau, China; 4 Department of Physiology, Yong Loo Lin School of Medicine, National University of Singapore, Singapore

**Keywords:** COVID-19, SARS-CoV-2, HCQ, CQ, therapy, clinical trials

## Abstract

The outbreak of coronavirus disease-19 (COVID-19) caused by severe acute respiratory syndrome coronavirus 2 (SARS-CoV-2) has rapidly evolved into a global pandemic. One major challenge in the battle against this deadly disease is to find effective therapy. Due to the availability and proven clinical record of hydroxychloroquine (HCQ) and chloroquine (CQ) in various human diseases, there have been enormous efforts in repurposing these two drugs as therapeutics for COVID-19. To date, substantial amount of work at cellular, animal models and clinical trials have been performed to verify their therapeutic potential against COVID-19. However, neither lab-based studies nor clinical trials have provided consistent and convincing evidence to support the therapeutic value of HCQ/CQ in the treatment of COVID-19. In this mini review we provide a systematic summary on this important topic and aim to reveal some truth covered by the mystery regarding the therapeutic value of HCQ/CQ in COVID-19.

## Introduction

The outbreak of COVID-19 caused by SARS-CoV-2 has caused a global public health emergency and serious economic crisis. Since December 2019, it has rapidly spread around the world and affected more than 200 countries and regions. According to WHO statistics, the outbreak of COVID-19 has caused more than 100 million confirmed cases and 2 million deaths. Globally there is an urgent demand for effective and affordable therapies and vaccines for the treatment and prevention, respectively.

The endo-lysosomal network is particularly important for the entry of coronavirus (CoV) into host cells, and this pathway has become an attractive therapeutic target for the development of antiviral agents 1. HCQ/CQ have a variety of pharmacological activities, and the mechanism of their anti-CoV (including SARS-CoV-2) effect has not been fully elucidated. Current studies have shown that HCQ/CQ can prevent CoVs from recognizing the receptor, inhibiting the acidification of endosomes that interferes with membrane fusion, and suppressing immunomodulatory process 2. Furthermore, HCQ/CQ promote the production of immunosuppressive factors IL-6 and TNFα and activate p38 MAPK 3. These mechanisms may work together to exert their therapeutic effect on SARS-CoV-2. A number of *in vitro* studies have shown that HCQ/CQ are effective on several types of CoV that causing human diseases, such as severe acute respiratory syndrome coronavirus (SARS-CoV) 4, Middle East respiratory syndrome coronavirus (MERS-CoV) 5, and SARS-CoV-2 6-8. However, in animal models, HCQ/CQ treatment could aggravate infection and cause unfavorable side-effects 9. More importantly, HCQ/CQ have shown mixed results in recent clinical trials: some with certain therapeutic benefits on the treatment of COVID-19 10-14; while others demonstrated the opposite effects: significant side effects and increased mortality 11, 15.

Some recent reviews have demonstrated the possible molecular mechanism of the anti-SARS-CoV-2 effect of HCQ/CQ 16, 17, evaluated the pharmacokinetics 18 and toxicity 19 of HCQ/CQ in clinical investigations. In this mini-review, we will summarize the current investigations of HCQ/CQ on SARS-CoV-2 by focusing on *in vitro* studies, animal models and clinical trials to evaluate the therapeutic potential of HCQ/CQ in the treatment of COVID-19, aiming to clear the mystery that currently cover the truth of HCQ/CQ as potential therapeutics for treatment of COVID-19.

## History of HCQ/CQ as therapeutics for malaria

HCQ/CQ have been extensively studied and widely used for the prevention and treatment of malaria for many decades. Quinine, a natural product extracted from the bark of the cinchona tree 20, was first documented to treat malaria in the 17^th^ century 21. Due to the insufficient supply of quinine in the early 19^th^ century, CQ was synthesized as a candidate substitution for quine as an anti-malaria drug in 1930s 22. Despite its effective anti-malaria function, CQ was not scaled up for clinical use because of its extreme toxicity. In 1940s, CQ was subsequently resynthesized by the introduction of a hydroxyl group, which was named as HCQ and this defined compound had been shown to be more active but less toxic than CQ 23. HCQ was first approved in 1955, with a favorable efficacy and a reduced toxicity, compared to CQ 24. Since then, both HCQ and CQ had been widely used as the first-line anti-malaria drugs.

In addition to the common usage of HCQ and CQ as anti-malaria drugs, they have been increasingly studied for their repurposing potentials in the treatment of an array of other diseases, such as rheumatoid arthritis, systemic lupus erythematosus, cancer, and other bacterial or viral infectious diseases 25-27. Notably, since the outbreak of the current COVID-19 pandemic, the antiviral effects and applications of HCQ and CQ in the treatment of COVID-19 have attracted particular interest.

## Effects of HCQ/CQ on the endocytic pathway in the host cells

**Figure 1:**
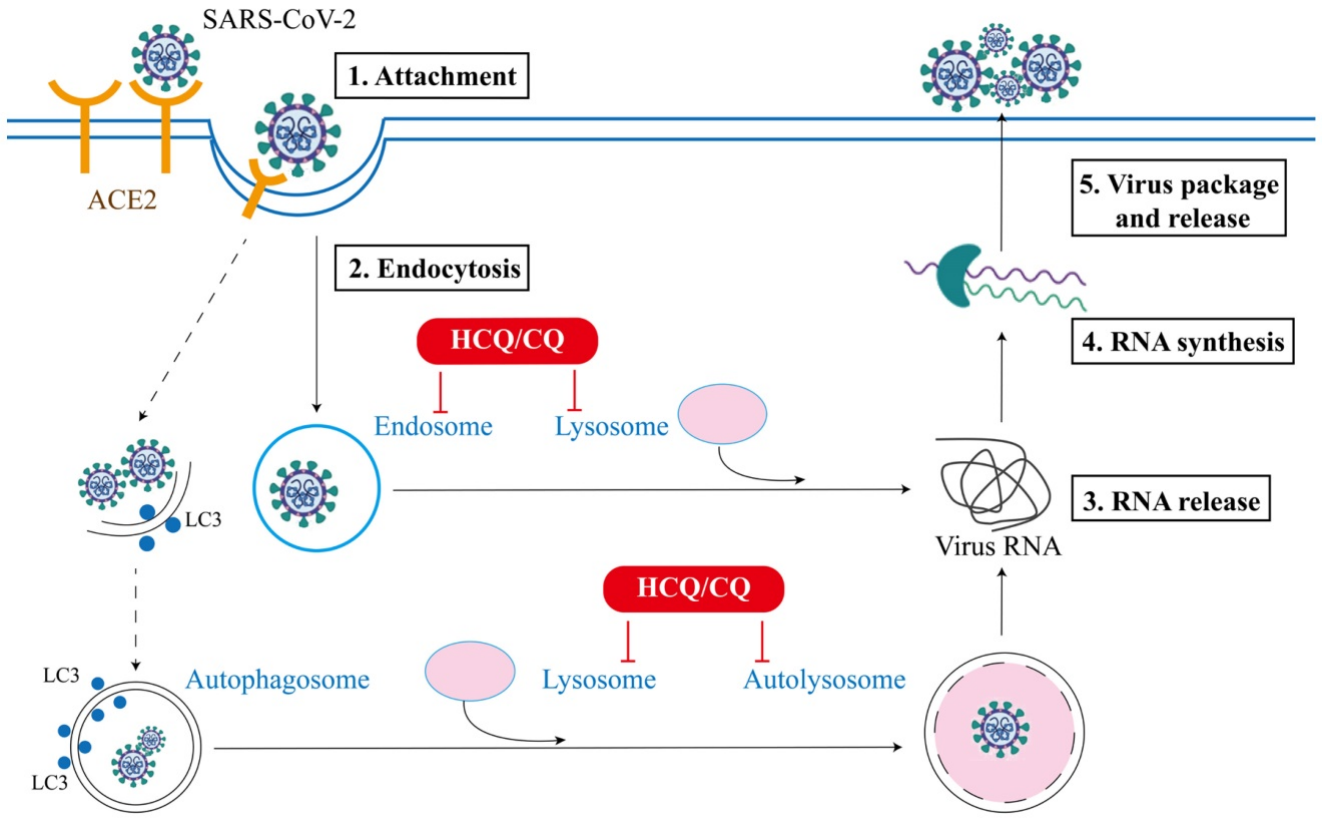
Schematic of SARS-CoV-2 attachment, entry, RNA release and synthesis, and virus assembly and release in the extracellular space. The endocytic pathway plays a critical role in the entry and replication of SARS-CoV-2 and serves as target for the potential therapeutic effects of HCQ/CQ in COVID-19.

SARS-CoV-2 is an enveloped virus with a positive-sense single-stranded RNA genome, and the viral envelope is coated by spike (S) protein trimers that bind to angiotensin converting enzyme 2 (ACE2) receptor, which is required for SARS-CoV-2 infection on host cells 28. To date, the main pathway controlling the entry of virus into the host cells is endocytosis 29. The endocytic machinery includes several processes, such as macropinocytosis, clathrin-dependent or clathrin-independent endocytosis, caveolae-dependent endocytosis or caveolae-independent endocytosis 30. As shown in **Figure 1**, the replication cycle of SARS-CoV-2 consists of the following 5 steps 31: (i) Attachment: the virus attach to the host receptor ACE2 via the viral spike protein, which will facilitates its internalization; (ii) Endocytosis: viral membrane fuses with the host cell membrane and gets entry via the host endocytic pathway (endosomes and lysosomes); (iii) Release of viral RNA: the internalized virus releases its genome into the cytosol to be replicated (lysosomes); (iv) Synthesis of viral RNA: genomic RNA experiences transcription and translation to produce relevant viral proteins; (v) Package and release: the viral components assemble together to form new virion which exit to extracellular space through exocytosis. Among them, the endocytic pathway is known to be implicated in the internalization, synthesis and the release stages of viral replication 32. Although the entry mechanisms and the implication of these routes in SARS-CoV-2 infection have not been fully understood, substantial studies have established that the endocytic pathway is the key mechanism controlling the entry of CoVs into the host cells, which usually depends on the low pH of endocytic organelles, including both endosomes and lysosomes 1. Thus, antiviral therapies have been developed to limit the spreading of viruses through blocking any one or combination of above steps of virus life cycle 33.

In addition to the endocytic pathway, lysosome also plays a critical role in the maturation stage of autophagy, by fusing with autophagosome and degrading the engulfed contents in the autophagosome 34. At present, the role of autophagy in viral infection has been widely investigated, while the crosslinks between these two processes are still controversial 1, 35. Earlier studies have demonstrated that autophagy is implicated in the formation of double-membrane vesicles (DMVs) as well as in the replication of mouse hepatitis virus (MHV) and SARS-CoV 36. In the process of virus infection, autophagy plays either a pro-viral or anti-viral role, depending on the type of virus, experimental approaches, and the cellular environment 36, 37. Since the outbreak of COVID-19 more than a year ago, there are some studies focusing on the interplays between the autophagy machinery and SARS-CoV-2: the autophagic machinery can exert either positive or negative effect on the replication cycle of SARS-CoV-2 and the viral proteins from SARS-CoV-2 have reciprocal effects on the autophagic process 35, 38. Such an intricate relationship between autophagy and SARS-CoV-2 lays the foundation for the potential therapeutic activity of HCQ/CQ in treatment of COVID-19.

## Antiviral effects of HCQ/CQ on SARS-CoV-2* in vitro*

Since the COVID-19 outbreak, the potential anti-viral effects of HCQ/CQ have been tested in various *in vitro* systems, as summarized in **Table 1**. For instance, there were experimental data demonstrating that CQ was effective in the control of SARS-CoV-2 infection *in vitro,* compared with other anti-viral agents, including ribavirin, penciclovir, nitazoxanide, nafamostat and favipiravir 39. Time-of-addition assay demonstrated that CQ could interfere with both entry and post-entry phases of the SARS-CoV-2 infection in Vero E6 cells, and authors implied that CQ is able to synergistically modulate immune activity to improve its antiviral activity 39. Furthermore, another study also demonstrated that CQ could block SARS-CoV-2 replication in a concentration-dependent manner while had little toxicity on the host cells 40. In a follow-up study of an *in vitro* work described above 39, authors found that HCQ was effective in inhibiting SARS-CoV-2 infection *in vitro* 41; HCQ also could block the entry and post-entry stages of SARS-CoV-2 confirmed by time-of-addition assay, which was also similarly found upon CQ treatment; moreover, both CQ and HCQ inhibit the release of the viral genome which depends on the transport of SARS-CoV-2 along the endocytic pathway 41.

HCQ has also demonstrated to be more effective but less toxic than CQ, suggesting that HCQ could be a better pharmacological candidate for treatment of COVID-19 (**Table 1**). After SARS-CoV-2 infection, the EC50 for CQ is higher than the EC50 for HCQ in Vero E6 cells 6. Moreover, treatment with HCQ, but not CQ, caused the improvement on the size and number of endolysosomes, 41. All these evidences indicate that the *in vitro* anti-SARS-CoV-2 efficacy of HCQ could be better than that of CQ.

## Effects of HCQ/CQ on SARS-CoV-2 in animal models

Various animal models have been utilized to evaluate the therapeutic effect of HCQ/CQ on SARS-CoV-2 infection. Researchers created non-human primate (cynomolgus macaque) models of SARS-CoV-2 infection to investigate the therapeutic potential of HCQ 43. In their study, the authors found that there is no obvious anti-SARS-CoV-2 effect of HCQ in the initiation of treatment, neither before infection, early after infection (before the peak of the viral load) nor late after infection (after the peak of the viral load). This conclusion was supported by several other animal works. For instance, the experimental result from SARS-CoV-2-infected Syrian hamsters illustrated that HCQ is unlikely to have anti-SARS-CoV-2 activity in this animal model, while favipiravir exhibits remarkable protective effect 44. Furthermore, ferret infection model was also used to assess the antiviral candidates of SARS-CoV-2, and data indicated that HCQ/CQ have no benefit for the improvement of SARS-CoV-2-infected animals, while the adverse effects such as heart rhythm need to take more concern 45.

Up to date, due to the biological safety issue and strict regulation applied for the personnel and facilities handling SARS-CoV-2, both the *in vitro* work and animal study on SARS-CoV-2 are still rather limited. There is an urgent need to establish more animal models with SARS-CoV-2 infection for testing the potential therapeutics including HCQ and CQ in the combat against the still expanding pandemic.

## Clinical trials with HCQ/CQ in COVID-19 patients

Since HCQ/CQ have been used on malaria therapy for several decades, the rationale and safety of these two agents in clinical administration are well established. Based on the well-known role of the endocytic pathway and autophagy in the infection process of SARS-CoV-2 as discussed above, the potential therapeutic efficacy of HCQ/CQ in COVID-19 has undergone extensive clinical testing all over the world, some of the main findings are summarized in **Table 2**. The efficacy of HCQ or CQ has been tested in about 10 hospitals in China and the results obtained from more than 100 COVID-19 patients confirmed the benefits of HCQ/CQ treatment compared with the control group by inhibiting the exacerbation of pneumonia, promoting a virus negative conversion, and shortening the disease course, without obvious side effects 46. Therefore, some countries such as China has included CQ in the recommendations regarding the prevention and treatment of COVID-19. However, the results from other clinical trials are not convincing and their therapeutic effects are unsatisfactory, as discussed in details below.

### HCQ/CQ in treatment of COVID-19

Currently, HCQ and CQ are among most widely studied antiviral drugs evaluated for SARS-CoV-2 treatment in more than one hundred clinical trials worldwide (https://www.clinicaltrials.gov/ct2/results?recrs=&cond=Covid19&term=chloroquine&cntry=&state=&city=&dist=). Once completed, the truth regarding the real therapeutic effects of HCQ/CQ might emerge from the mystery.

One clinical trial was conducted in Guangdong China to evaluate the therapeutic potential of CQ on COVID-19 patients. The results suggested that CQ could be an effective and available option among current proposed therapies in the patients with mild/general SARS-CoV-2 infection 10. In this study, CQ-treated patients appeared to regain their pulmonary function quicker and get sooner recovery than those patients treated with other antiviral agents (lopinavir/ritonavir, a protease inhibitor combination treatment for human immunodeficiency virus (HIV) infection 47). Results from another clinical study claimed that both CQ and HCQ could promote viral RNA negativity and reduce the time to clinical recovery (TTCR) in moderate form of COVID-19 48; one randomized controlled trial using HCQ for treatment of COVID-19 was conducted in Wuhan China and the results shown that HCQ treatment meliorated the fever and reduced the cough duration 49, suggesting that HCQ may be a potential treatment for critically COVID-19 patients. More importantly, one clinical study with critical ill COVID-19 patients demonstrated that HCQ even could significantly reduce death risk without apparent side-effect on COVID-19 patients 12.

Despite the positive results from the clinical studies as discussed above, more clinical evidence has challenged the therapeutic efficacy of HCQ/CQ on COVID-19 patients and some of clinical trials are summarized in **Table 2.** First, there were no significant differences in clinical symptoms, inflammatory biomarkers, length of hospital stay and overall mortality between control group and HCQ/CQ group 50-52 while the administration of HCQ even increased the risk of mortality 53. Second, high-dose CQ was associated with the incidence of gastrointestinal disorder, cardiovascular lethality and QTc interval 11, 14, 54, 55, which will be discussed in details below.

There are several explanations for the ineffectiveness of HCQ/CQ in the treatment of COVID-19 patients. A recent study by Hadjadj et al. found that patients with severe COVID-19 had impaired IFN-I activity, increased T cell apoptosis and inflammatory response, while the regulatory effect on immune response by HCQ/CQ is not potent enough to inhibit the over-activation of innate immune system 56; another analysis suggested that the ORF3a of SARS-CoV-2 could block the fusion between autophagosomes and lysosomes, so SARS-CoV-2 can survive by escaping lysosome destruction 57, which made the role of autophagy-lysosome more complicated in the living cycle of SARS-CoV-2.

### Combinational therapy of HCQ with azithromycin in treatment of COVID-19

In order to improve the clinical therapeutic effect of current antiviral agents, one important approach is to use combinational therapy. Among various combinations, a couple of clinical studies have been carried out on the combination of HCQ and azithromycin (AZ). AZ is a synthetic macrolide antibiotic effective against a wide range of bacterial and mycobacterial infections, which has been prescribed to patients infected with SARS-CoV previously 59. Lately, AZ has been identified as a potential candidate for the treatment of SARS-CoV-2, and its effect has been evaluated by *in vitro*, in silico drug screens and clinical trials 60. However, the synergistic effect of HCQ and AZ in the treatment of SARS-CoV-2 is still questionable, as summarized in **Table 3**.

In France, one clinical trial with 36 participants indicated that combination of HCQ with AZ demonstrated a better efficacy than single drug, based on the virus clearance rate 61. However, majority studies indicated that the combination of HCQ and AZ could not take a favorable turn of the course of SARS-CoV-2 patients. In a retrospective multicenter cohort study on totally 1,438 SARS-CoV-2 patients from 25 different hospitals in New York, analysis data revealed that the hospital fatality rate among different treatment groups did not show significant difference 62. Consistently, a retrospective analysis of 368 patients from USA suggested that risk of ventilation had no significant difference in HCQ+AZ group from control group 63. Whereas two studies consistently claimed that the combination of HCQ and AZ caused unfavorable side-effects, including heart failure and cardiovascular fatality within 30-days treatment period in COVID-19 patients 64, 65.

One systematic review and meta-analysis published in January 2021 indicated that the combination of HCQ and AZ increased mortality significantly, summarized from 29 articles, including randomized controlled trials, non-randomized trials and observational studies 66. In conclusion, majority of current clinical trials on this combined treatment have not provided any optimistic outcome for their further application on COVID-19 treatment, mainly because of their ineffectiveness on COVID-19 and significant advert effects on cardiovascular system. Therefore, strict cardiovascular monitoring should be applied if this regimen is given.

### Side effects of HCQ/CQ in clinical trials for treatment of COVID-19

Although the application of HCQ/CQ is well established in malaria or autoimmune diseases for years, higher dosage seems to be a prerequisite for the anti-viral effects of HCQ/CQ against SARS-CoV-2. According to the clinical reports, suggested doses of HCQ/CQ in COVID‐19 patients are significantly higher than the recommended dose for malaria treatment 68. Significant side effects have been reported from the clinical investigations, such as anxiety, sleeplessness, gastrointestinal disorders, and cardiomyopathy, as summarized in both **Table 2** and **Table 3**.

The most common side effects of HCQ/CQ were gastrointestinal responses, such as vomiting and diarrhea, which widely affect the recovery of patients 20, 49, 54, 58. Long-term administration of these two drugs would cause plenty of adverse effects, such as retinopathy, circular defects, diametric defects in the retina and cardiomyopathy 27, 55. COVID-19 patients could be more vulnerable to side effects, particularly among the high‐risk patients group: the elderly and patients with chronic diseases, such as diabetes or cardiovascular diseases. In addition, cardiotoxicity caused by CQ seems specifically relevant to the infection of SARS-CoV-2. For instance the myocarditis is a very common complication after infecting SARS-CoV-2, especially in the combination with QTc interval prolonging drugs, for example amiodarone, macrolide antibiotics (such as AZ), ondansetron, and many others 41. Likewise, the notable QTc interval prolongation (>500ms) with a high risk for arrhythmia was found in the case of co-treatment with HCQ and AZ 65, 67, 69. Both HCQ and CQ are mainly metabolized via the liver and kidney and have long half-lives (approximately 1-2 months) 70. Therefore, long-term monitoring for their renal and hepatotoxicity is necessary.

Although HCQ and CQ share similar chemical structures and both of them have potential anti-SARS-CoV-2 activity as discussed earlier, results from some clinical trials suggest that HCQ has fewer side effects and higher tolerated doses in comparison to CQ 71. Thus, CQ has more restrictions in treatment of COVID-19 as CQ overdose can rapidly cause sever life-threaten toxicity, corresponding to a dose of 5 g in adults could result in death without treatment 72. There two major reasons have been proposed for this: (i) The maximum tolerable dose for HCQ is 1,200 mg, which has an antiviral effect equivalent to 750 mg CQ (for which the maximum tolerable dose is 500 mg) 73; (ii) CQ could exert various advert effects on fetal development, therefore, HCQ may be a safer option comparing to CQ, for the pregnant women with COVID-19 74, and data from rheumatological research indicates that CQ is linked to a higher retinopathy incidence rate when compared with HCQ 75.

In conclusion, it is important to understand the exact antiviral mechanism of HCQ/CQ, then optimize the application of HCQ/CQ in battling against SARS-CoV-2 while consider the toxicological risks and necessary care for patients after drug administration.

## Summary and Perspectives

Given the COVID-19 global pandemic, there is an urgent need for effective and available antiviral therapy and vaccines. Despite the long history of clinical application of HCQ and CQ in various human diseases, with advantages of inexpensive and easily accessible, their therapeutic value in treatment of COVID-19 remains questionable. The mystery or controversy comes from several aspects. First, both CQ and HCQ could inhibit the transport of SARS-CoV-2 along the endocytic pathway via neutralizing the pH value of acidic organelles (endosome and lysosome) in host cells, which have been verified by several *in vitro* studies. Second, insufficient and controversial results from SARS-CoV-2 animal models, which greatly challenged the *in vivo* antiviral effect of HCQ/CQ. Third, most clinical trials failed to prove the efficacy of HCQ/CQ on COVID-19 patients but discover obvious cardiovascular toxicity and gastrointestinal responses from various clinical trials. As a result, in June 2020 FDA revoked its authorization for the emergency use of HCQ/CQ in COVID-19 patients (https://www.fda.gov/news-events/press-announcements/coronavirus-covid-19-update-fda-revokes-emergency-use-authorization-chloroquine-and).

At present, there are still numerous clinical trials ongoing around the world using HCQ/CQ in treatment of COVID-19, either alone or in combination with other therapeutics. To move forward, there are important challenges for the scientific community to conduct more work to repurpose these two ancient drugs in the combat against this deadly COVID-19 pandemic. Specifically, more mechanistic studies are necessary to fully discover the exact targets of HCQ/CQ on both SARS-CoV-2 and host cells, to clarify the potential role of autophagy and lysosome on the process of viral replication. Furthermore, more animal works are needed to reveal the pharmacokinetic characteristics of HCQ/CQ and understand the possible reason for the inconsistent effect of these two agents between *in vitro* and *in vivo* investigations. Last and most importantly, it will be critically important to conduct more clinical trials to optimize the clinical application, including potential combined therapy, to enhance the therapeutic efficacy and to reduce the adverse effects on patients. Hopefully, all the research work not only resolve the mystery regarding the therapeutic efficacy of these two drugs in COVID-19, also add more light at the end of tunnel in our fight against COVID-19.

## Figures and Tables

**Table 1:** HCQ/CQ as antiviral agents* in vitro*

Agents	Infected cells	Dose range	Mechanisms	Main findings	Ref
HCQ/CQ	Vero E6 cells	50 μM for 1 hr	Elevation of the pH of lysosome/endosome that inhibits SARS-CoV entry and post-entry stage	HCQ and CQ exerted the anti-SARS-CoV-2 effect; HCQ was safer than CQ in the treatment of SARS-CoV-2 infection	41
HCQ/CQ	Vero E6 cells	0.032-100 μM for 24 or 48 hrs.	N. A	HCQ/CQ had inhibitory effect on SARS-CoV-2; the anti-SARS-CoV-2 activity of HCQ was more potent than CQ.	6
CQ	Vero E6 cells	10 μM for 48 hrs	Inhibition of the entry, and post-entry stage of the SARS-CoV-2 infection	CQ was effective to protect against the spread of SARS-CoV-2	39
CQ	Vero E6 cells and HEK293T cells	1-10-100 μM for 1 hr	Inhibition of the TMPRSS2 and CTSL-dependent entry of SARS-CoV-2	CQ had no effect in the control of SARS-CoV-2 infection *in vitro*	42

CQ, chloroquine; HCQ, hydroxychloroquine; CTSL, cathepsin L; N.A, not applicable

**Table 2:** Clinical trials and retrospective studies using HCQ/CQ in treatment of COVID-19

Agents	Type of Study	Design of Treatment	Main Findings	Side Effects	Ref
HCQ/CQ	Phase I clinical trial (open-label, randomized controlled trial)	n=48; 18 in CQ group, 18 in HCQ group and the rest in control group	Both HCQ and CQ decreased the time to viral RNA negativity, TTCR and duration of hospitalization	Diarrhea	48
HCQ	Retrospective study	n=550; 502 received basic treatments; 48 received HCQ 200mg/day, 7-10 days twice daily	HCQ significantly reduces death risk of critically COVID-19 patients without apparent toxicity	N. A	12
HCQ	Phase I clinical trial (randomized-clinical trial)	n=62; 31 in the standard treatment group; 31 in the HCQ-treated group (400 mg/day, 5 days)	Shorten the TTCR and promoted the absorption of pneumonia.	Diarrhea, nausea, fatigue, chest tightness	49
					
HCQ	Phase Ⅱ clinical trial	n=211; received HCQ after post-exposure prophylaxis (PEP)	After PEP using HCQ, PCR tests of all individuals were negative	Diarrhea or loose stool, skin rash and bradycardia	54
HCQ	Phase I clinical trial (multicenter, randomized, open-label, controlled trial)	n=33; 21 in HCQ group, 12 in the standard of care treatment group	No significant clinical benefit of HCQ was observed	Headache, dizziness. gastritis, diarrhea, nausea and photophobia	14
HCQ	Retrospective analysis	n=37; 28 in HCQ group, 9 in the standard of care treatment group	No significant clinical benefit of HCQ was observed	N. A	14
HCQ	Phase Ⅱ clinical trial (multicenter, randomized-controlled trial)	n=194; 97 in HCQ group, 97 in control group	No significant differences in clinical outcomes and overall mortality	N. A	50
HCQ	Phase Ⅱ clinical trial (Randomized controlled open label trial)	n=89; 44 in HCQ group; 45 in the favipiravir + interferon β-1b group	No significant differences in clinical signs and symptoms, inflammatory biomarkers, length of hospital stay, and mortality rate. HCQ has no proven clinical effects	N. A	51
HCQ	Phase Ⅱ clinical trial (open-label and randomize-controlled clinical trial)	n=293; 157 received HCQ (800 mg once, followed by 400 mg, 6 days once daily), the rest received standard care	No significant differences in the risk of hospitalization and time to recover from symptoms	N. A	52
HCQ	Retrospective analysis	n=1,669; 696 in HCQ group, 973 in treatment without HCQ group	HCQ had no benefit on mortality in COVID-19 patients.; but increased the risk of mortality	N. A	53
HCQ	Retrospective analysis	n=60; 30 in febuxostat group, 30 in HCQ group	No significant efficacy in clinical symptoms	Retinal toxicity, cardiac toxicity, QTc interval prolongation	55
HCQ	Phase Ⅱ clinical trial (randomized, double-blind, placebo-controlled trial)	n=821; assigned equally into 2 groups: HCQ (800 mg in 6 to 8 hours, then 600 mg daily for 4 additional days) and placebo	The incidence of COVID-19 had no significant difference	Nausea, loose stools, abdominal discomfort	58
CQ	Phase I randomized clinical trial	n=22; 10 in CQ 500 mg/day, twice-daily for 10 days; 12 in Lopinavir/Ritonavir 400/100 mg, twice-daily for 10 days	Patients treated with CQ regained their pulmonary function quicker and recovered sooner	No serious adverse events	10
CQ	Phase Ⅱ clinical trial	n=81; 41 in CQ high-dosage group (600 mg /day, 10 day twice daily), 40 in the CQ low-dosage group (450 mg/day, 10 day, twice daily)	No clinical benefit of CQ was observed, and high-dose CQ associated with lethality and QTc interval	QTc interval prolongation	11

CQ, chloroquine; HCQ, hydroxychloroquine; COVID-19, novel coronavirus infection 2019; IL-6, interleukin-6; PCR, polymerase chain reaction; ICU, Intensive Care Unit; CT, Computer Tomography; CRP, curved planar reformation; COVID-19, novel coronavirus infection 2019; rRT-PCR, reverse real-time polymerase chain reaction; N.A; not applicable; PEP, post-exposure prophylaxis; TTCR, time to clinical recovery.

**Table 3:** Clinical trials and retrospective studies on combinational therapy of HCQ with AZ in treatment of COVID-19

Agents	Type of study	Design of Treatment	Main Findings	Side effects	Ref
HCQ + AZ	Phase I clinical trial (open-label non-randomized)	n=42; 26 received HCQ+AZ (600 mg/day), 16 were control group	HCQ treatment associated with viral load reduction or disappearance; this effect is reinforced by AZ	N. A	61
HCQ + AZ	Retrospective study	n=1,438; 735 in HCQ+AZ group, 271 in HCQ group. 221 in AZ group, 221 in control group.	Abnormal electrocardiogram and in-hospital mortality had no significant difference from control group	N. A	62
HCQ + AZ	Retrospective study	n=368; 97 in HCQ group, 113 in HCQ+AZ group, 158 in no HCQ group	No reduced risk of mechanical ventilation after treatment	N. A	63
HCQ + AZ	Retrospective study	n=251; All patients received HCQ+AZ, HCQ was given at 400 mg once followed by 200 mg 4 days twice daily, AZ 500 mg/day, once daily for 5 days	QTc interval prolongation and induction of torsade de pointes, strict QTc interval monitoring should be applied if this regimen is given	QTc interval prolongation and induction of torsade de pointes	65
HCQ + AZ	Cohort study	n=1,941,802; 956,374 in HCQ group and 310,350 in sulfasalazine group, 323,122 in HCQ+AZ group and 351,956 in HCQ+amoxicillin group	The combination of AZ and HCQ increased the risk of heart failure and cardiovascular mortality	Angina/chest pain; heart failure; cardiovascular mortality	67

CQ, chloroquine; HCQ, hydroxychloroquine; N.A, not applicable; AZ, azithromycin.

## Abbreviations

AZ: azithromycin
ACE2: angiotensin converting enzyme 2
CoV: coronavirus
COVID-19: coronavirus disease 2019
CQ: chloroquine
CRP: curved planar reformation
CT: computer tomography
CTSL: cathepsin L
DMVs: double-membrane vesicles
HCQ: hydroxychloroquine
HIV: human immunodeficiency virus
ICU: intensive care unit
IL-6: interleukin-6
MERS-CoV: Middle East respiratory syndrome coronavirus
MHV: mouse hepatitis virus
PCR: polymerase chain reaction
rRT-PCR: reverse real-time polymerase chain reaction
PEP: post-exposure prophylaxis
SARS-CoV: severe acute respiratory syndrome coronavirus
SARS-CoV-2: severe acute respiratory syndrome coronavirus 2
TTCR: time to clinical recovery
